# 6-(3,4-Di­fluoro­phen­yl)-7,8,13,14-tetra­hydro­dibenzo[*c*,*k*]phenanthridine

**DOI:** 10.1107/S2414314620012419

**Published:** 2020-09-11

**Authors:** Yiwen Fang, Bingbing Liu, Zhixiang Jia

**Affiliations:** a Department of Chemistry, Anhui University, Hefei, Anhui 230039, People’s Republic of China; b Department of Chemistry, Yancheng Teachers College, Yancheng, Jiangsu, 224002, People’s Republic of China; University of Aberdeen, Scotland

**Keywords:** crystal structure, 2-phenyl­pyridine, pyridine ring

## Abstract

In the title compound, C_27_H_19_F_2_N, the five-fused-ring system is highly puckered and the dihedral angle between the central pyridine ring and pendant di­fluoro­benzene ring is 45.12 (12)°. In the crystal, inversion dimers linked by pairwise weak C—H⋯N hydrogen bonds generate *R* ^2^_2_(12) loops and the dimers are further linked by weak C—H⋯F inter­actions to form [  01] chains.

## Abstract 2

In the title compound, the dihedral angle between the pyridine and di­fluoro­benzene rings is 45.12 (12)°.

## Graphical abstract

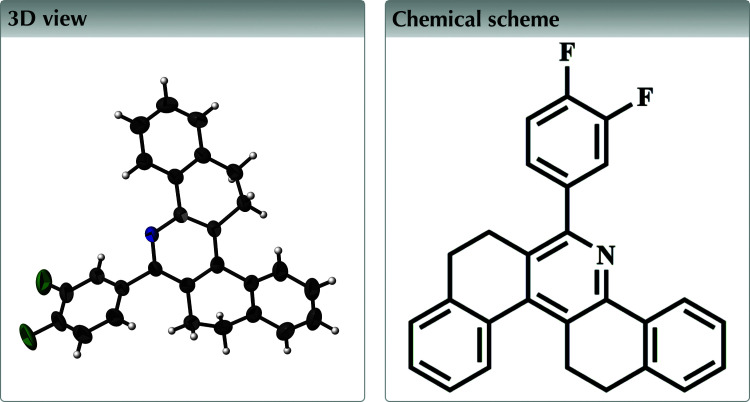

## Structure description

**Figure 1:**
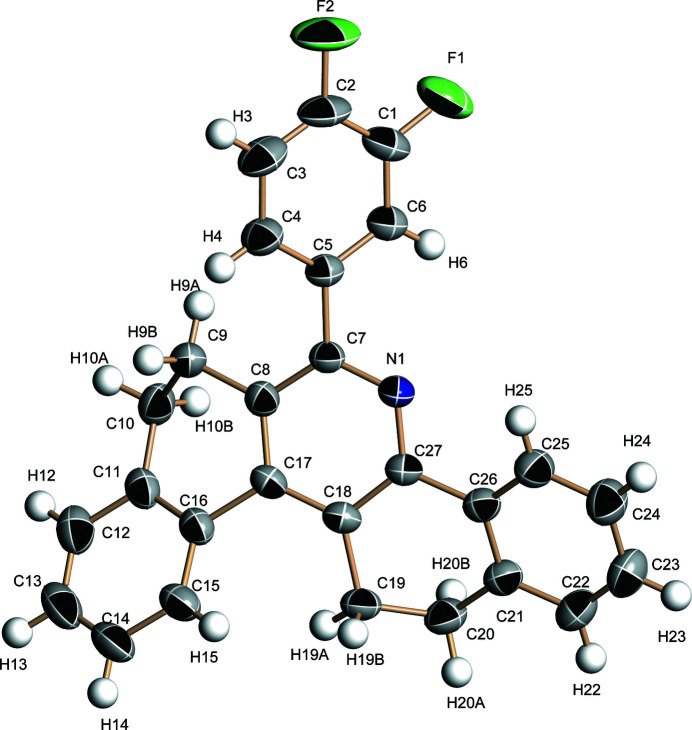
The mol­ecular structure of the title compound showing 50% displacement ellipsoids.

In recent years, nitro­gen-containing heterocyclic mol­ecular materials have found use as optoelectronic materials (Gu *et al.* 2017 ▸; Zhang *et al.*, 2019 ▸) because of their conjugated structures and photophysical properties. In this work, we describe the synthesis and structure of the title compound (Fig. 1 ▸) in which the F atoms should increase solubility and provide strong electron-withdrawing groups.

**Figure 2:**
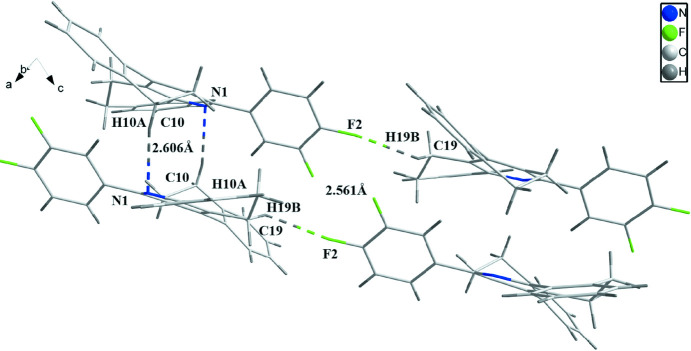
Partial packing diagram showing hydrogen bonds as dashed lines.

The crystal structure shows that the dihedral angle between the C1–C6 di­fluoro­benzene ring and the adjacent C7/C8/C17/C18/C27/N1 pyridine ring is 45.12 (12)°. In the crystal, pairwise weak C10—H10*B*⋯N1 hydrogen bonds (Table 1 ▸) link the mol­ecules into inversion dimers, which are further linked by weak C—H⋯F inter­actions (Fig. 2 ▸) to form [ 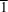 01] double chains.

## Synthesis and crystallization

Ammonium acetate (7.60 g, 0.100 mol) was dissolved in 15 ml glacial acetic acid. Then, 3,4-di­fluoro­benzaldehyde (2.00 g, 0.014 mol) and 1-tetra­hydro­naphthalone (4.12 g, 0.028 mol) were added and the reaction was heated to reflux at 383 K for 5 h. Upon cooling and recrystallization from ethanol solution, 1.64 g (yield 30%) of the title compound was recovered. Crystals for X-ray analysis were obtained from the slow evaporation of an aceto­nitrile solution.

## Refinement

Crystal data, data collection and structure refinement details are summarized in Table 2 ▸.

## Supplementary Material

Crystal structure: contains datablock(s) I. DOI: 10.1107/S2414314620012419/hb4358sup1.cif

Structure factors: contains datablock(s) I. DOI: 10.1107/S2414314620012419/hb4358Isup2.hkl

Supporting information file. DOI: 10.1107/S2414314620012419/hb4358Isup3.cml

CCDC reference: 2012681

Additional supporting information:  crystallographic information; 3D view; checkCIF report

## Figures and Tables

**Table 1:** Hydrogen-bond geometry (Å, °)

*D*—H⋯*A*	*D*—H	H⋯*A*	*D*⋯*A*	*D*—H⋯*A*
C10—H10*B*⋯N1^i^	0.97	2.61	3.552 (4)	165
C19—H19*A*⋯F2^ii^	0.97	2.56	3.111 (4)	116

Symmetry codes: (i) 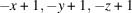 ; (ii) 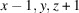 .

**Table 2:** Experimental details

Crystal data	
Chemical formula	C_27_H_19_F_2_N
*M*_r_	395.43
Crystal system, space group	Triclinic, *P* 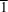
Temperature (K)	296
*a*, *b*, *c* (Å)	9.394 (5), 9.802 (5), 11.914 (6)
α, β, γ (°)	88.983 (5), 73.144 (5), 69.488 (5)
*V* (Å^3^)	979.0 (8)
*Z*	2
Radiation type	Mo *K*α
μ (mm^−1^)	0.09
Crystal size (mm)	0.03 × 0.02 × 0.01

Data collection	
Diffractometer	Bruker APEXII CCD area detector
Absorption correction	Multi-scan (*SADABS*; Bruker, 2013 ▸)
*T*_min_, *T*_max_	0.633, 0.746
No. of measured, independent and observed [*I* > 2σ(*I*)] reflections	7892, 4051, 3053
*R*_int_	0.027
(sin θ/λ)_max_ (Å^−1^)	0.651

Refinement	
*R*[*F* ^2^ > 2σ(*F* ^2^)], *wR*(*F* ^2^), *S*	0.063, 0.190, 1.06
No. of reflections	4051
No. of parameters	271
H-atom treatment	H-atom parameters constrained
Δρ_max_, Δρ_min_ (e Å^−3^)	0.43, −0.25

Computer programs: *APEX2* and *SAINT* (Bruker, 2013 ▸), *SHELXT* (Sheldrick, 2015*a* ▸), *SHELXL* (Sheldrick, 2015*b* ▸) and *OLEX2* (Dolomanov *et al.*, 2009 ▸).

## full crystallographic data

### Crystal data

**Table:**

C_27_H_19_F_2_N	*Z* = 2
*M**_r_* = 395.43	*F*(000) = 412
Triclinic, *P*1	*D*_x_ = 1.341 Mg m^−^^3^
*a* = 9.394 (5) Å	Mo *K*α radiation, λ = 0.71073 Å
*b* = 9.802 (5) Å	Cell parameters from 2895 reflections
*c* = 11.914 (6) Å	θ = 2.4–27.5°
α = 88.983 (5)°	µ = 0.09 mm^−^^1^
β = 73.144 (5)°	*T* = 296 K
γ = 69.488 (5)°	Block-shaped, white
*V* = 979.0 (8) Å^3^	0.03 × 0.02 × 0.01 mm

### Data collection

**Table:**

Bruker APEXII CCD area detector diffractometer	3053 reflections with *I* > 2σ(*I*)
phi and ω scans	*R*_int_ = 0.027
Absorption correction: multi-scan (SADABS; Bruker, 2013)	θ_max_ = 27.6°, θ_min_ = 1.8°
*T*_min_ = 0.633, *T*_max_ = 0.746	*h* = −12→12
7892 measured reflections	*k* = −12→10
4051 independent reflections	*l* = −15→15

### Refinement

**Table:**

Refinement on *F*^2^	Primary atom site location: dual
Least-squares matrix: full	Hydrogen site location: inferred from neighbouring sites
*R*[*F*^2^ > 2σ(*F*^2^)] = 0.063	H-atom parameters constrained
*wR*(*F*^2^) = 0.190	*w* = 1/[σ^2^(*F*_o_^2^) + (0.0837*P*)^2^ + 0.5466*P*] where *P* = (*F*_o_^2^ + 2*F*_c_^2^)/3
*S* = 1.06	(Δ/σ)_max_ < 0.001
4051 reflections	Δρ_max_ = 0.43 e Å^−^^3^
271 parameters	Δρ_min_ = −0.25 e Å^−^^3^
0 restraints	

### Special details

**Table:**

Geometry. All esds (except the esd in the dihedral angle between two l.s. planes) are estimated using the full covariance matrix. The cell esds are taken into account individually in the estimation of esds in distances, angles and torsion angles; correlations between esds in cell parameters are only used when they are defined by crystal symmetry. An approximate (isotropic) treatment of cell esds is used for estimating esds involving l.s. planes.

### Fractional atomic coordinates and isotropic or equivalent isotropic displacement parameters (Å^2^)

**Table:**

	*x*	*y*	*z*	*U*_iso_*/*U*_eq_	
N1	0.7467 (2)	0.3707 (2)	0.54946 (15)	0.0391 (4)	
F1	1.0054 (3)	0.3247 (2)	0.10698 (15)	0.0904 (6)	
F2	1.1593 (2)	0.5132 (3)	0.08204 (17)	0.1047 (8)	
C18	0.5333 (2)	0.4724 (2)	0.73116 (18)	0.0380 (5)	
C17	0.5140 (2)	0.6141 (2)	0.69776 (18)	0.0381 (5)	
C8	0.6055 (3)	0.6315 (2)	0.58465 (19)	0.0391 (5)	
C7	0.7235 (2)	0.5064 (2)	0.51581 (18)	0.0363 (5)	
C5	0.8364 (2)	0.5126 (2)	0.39951 (18)	0.0386 (5)	
C27	0.6517 (2)	0.3545 (2)	0.65376 (18)	0.0374 (5)	
C16	0.3991 (3)	0.7498 (2)	0.7746 (2)	0.0417 (5)	
C26	0.6825 (3)	0.2019 (2)	0.68702 (19)	0.0410 (5)	
C21	0.5609 (3)	0.1703 (3)	0.7718 (2)	0.0439 (5)	
C6	0.8684 (3)	0.4139 (3)	0.30477 (19)	0.0443 (5)	
H6	0.817554	0.346510	0.313138	0.053*	
C11	0.3292 (3)	0.8721 (3)	0.7200 (2)	0.0456 (6)	
C22	0.5900 (3)	0.0269 (3)	0.8019 (2)	0.0534 (6)	
H22	0.510292	0.005181	0.857526	0.064*	
C4	0.9152 (3)	0.6117 (3)	0.3848 (2)	0.0510 (6)	
H4	0.894687	0.678252	0.447433	0.061*	
C19	0.4315 (3)	0.4353 (3)	0.84374 (19)	0.0462 (6)	
H19A	0.328771	0.514474	0.870742	0.055*	
H19B	0.483188	0.426509	0.904573	0.055*	
C20	0.4060 (3)	0.2928 (3)	0.8250 (2)	0.0508 (6)	
H20A	0.350530	0.267914	0.899957	0.061*	
H20B	0.339713	0.306392	0.773529	0.061*	
C15	0.3651 (3)	0.7608 (3)	0.8972 (2)	0.0517 (6)	
H15	0.416490	0.682039	0.933526	0.062*	
C1	0.9756 (3)	0.4166 (3)	0.1989 (2)	0.0549 (7)	
C25	0.8280 (3)	0.0895 (3)	0.6371 (2)	0.0508 (6)	
H25	0.908948	0.109709	0.581439	0.061*	
C9	0.5619 (3)	0.7816 (3)	0.5415 (2)	0.0480 (6)	
H9A	0.595581	0.772922	0.456085	0.058*	
H9B	0.616434	0.836198	0.568047	0.058*	
C10	0.3802 (3)	0.8638 (3)	0.5889 (2)	0.0510 (6)	
H10A	0.352889	0.961687	0.563338	0.061*	
H10B	0.325601	0.812838	0.558290	0.061*	
C2	1.0529 (3)	0.5146 (3)	0.1853 (2)	0.0615 (8)	
C12	0.2169 (3)	0.9997 (3)	0.7903 (3)	0.0588 (7)	
H12	0.166393	1.080199	0.755108	0.071*	
C3	1.0245 (3)	0.6118 (3)	0.2769 (3)	0.0617 (7)	
H3	1.077498	0.677447	0.267400	0.074*	
C24	0.8549 (3)	−0.0521 (3)	0.6687 (3)	0.0618 (7)	
H24	0.953022	−0.126144	0.634986	0.074*	
C23	0.7334 (4)	−0.0818 (3)	0.7512 (3)	0.0617 (7)	
H23	0.749976	−0.176760	0.772106	0.074*	
C13	0.1814 (3)	1.0058 (3)	0.9111 (3)	0.0670 (8)	
H13	0.106587	1.090484	0.956823	0.080*	
C14	0.2552 (3)	0.8882 (3)	0.9651 (2)	0.0616 (8)	
H14	0.231630	0.894100	1.046662	0.074*	

### Atomic displacement parameters (Å^2^)

**Table:**

	*U*^11^	*U*^22^	*U*^33^	*U*^12^	*U*^13^	*U*^23^
N1	0.0375 (9)	0.0410 (10)	0.0340 (9)	−0.0146 (8)	−0.0032 (7)	0.0015 (7)
F1	0.0965 (14)	0.0996 (14)	0.0468 (9)	−0.0197 (11)	0.0020 (9)	−0.0195 (9)
F2	0.0783 (13)	0.1363 (19)	0.0616 (11)	−0.0315 (13)	0.0245 (10)	0.0223 (11)
C18	0.0354 (11)	0.0462 (12)	0.0301 (10)	−0.0133 (9)	−0.0084 (8)	0.0024 (9)
C17	0.0362 (11)	0.0438 (12)	0.0323 (10)	−0.0126 (9)	−0.0093 (9)	−0.0029 (9)
C8	0.0441 (12)	0.0381 (12)	0.0367 (11)	−0.0150 (9)	−0.0142 (9)	0.0026 (9)
C7	0.0363 (11)	0.0404 (12)	0.0321 (10)	−0.0161 (9)	−0.0073 (9)	0.0031 (8)
C5	0.0335 (10)	0.0440 (12)	0.0359 (11)	−0.0130 (9)	−0.0086 (9)	0.0076 (9)
C27	0.0356 (11)	0.0430 (12)	0.0322 (10)	−0.0146 (9)	−0.0080 (9)	0.0039 (9)
C16	0.0388 (11)	0.0412 (12)	0.0435 (12)	−0.0159 (10)	−0.0079 (10)	−0.0038 (9)
C26	0.0436 (12)	0.0450 (13)	0.0365 (11)	−0.0182 (10)	−0.0126 (9)	0.0065 (9)
C21	0.0465 (13)	0.0485 (13)	0.0405 (12)	−0.0211 (11)	−0.0141 (10)	0.0079 (10)
C6	0.0407 (12)	0.0491 (13)	0.0385 (12)	−0.0128 (10)	−0.0096 (10)	0.0056 (10)
C11	0.0409 (12)	0.0415 (12)	0.0525 (13)	−0.0166 (10)	−0.0086 (10)	−0.0058 (10)
C22	0.0581 (15)	0.0546 (15)	0.0588 (15)	−0.0317 (13)	−0.0212 (13)	0.0177 (12)
C4	0.0481 (14)	0.0571 (15)	0.0507 (14)	−0.0268 (12)	−0.0098 (11)	0.0074 (11)
C19	0.0460 (13)	0.0494 (14)	0.0342 (11)	−0.0139 (11)	−0.0030 (10)	0.0051 (10)
C20	0.0458 (13)	0.0538 (15)	0.0490 (14)	−0.0199 (11)	−0.0068 (11)	0.0125 (11)
C15	0.0518 (14)	0.0596 (16)	0.0416 (12)	−0.0247 (12)	−0.0048 (11)	−0.0093 (11)
C1	0.0470 (14)	0.0653 (16)	0.0347 (12)	−0.0045 (12)	−0.0055 (10)	0.0015 (11)
C25	0.0453 (13)	0.0473 (14)	0.0509 (14)	−0.0126 (11)	−0.0068 (11)	0.0055 (11)
C9	0.0611 (15)	0.0432 (13)	0.0378 (12)	−0.0214 (11)	−0.0088 (11)	0.0036 (10)
C10	0.0601 (15)	0.0402 (13)	0.0547 (14)	−0.0169 (11)	−0.0221 (12)	0.0105 (11)
C2	0.0416 (13)	0.0790 (19)	0.0458 (14)	−0.0149 (13)	0.0038 (11)	0.0188 (13)
C12	0.0491 (14)	0.0445 (14)	0.0769 (19)	−0.0148 (12)	−0.0126 (13)	−0.0036 (13)
C3	0.0443 (14)	0.0709 (18)	0.0691 (18)	−0.0283 (13)	−0.0078 (13)	0.0229 (15)
C24	0.0564 (16)	0.0481 (15)	0.0728 (18)	−0.0126 (12)	−0.0157 (14)	0.0090 (13)
C23	0.0649 (17)	0.0479 (15)	0.0777 (19)	−0.0218 (13)	−0.0285 (15)	0.0190 (13)
C13	0.0520 (15)	0.0613 (18)	0.0717 (19)	−0.0205 (14)	0.0065 (14)	−0.0248 (15)
C14	0.0593 (16)	0.0686 (18)	0.0491 (14)	−0.0277 (14)	0.0024 (12)	−0.0188 (13)

### Geometric parameters (Å, º)

**Table:**

N1—C7	1.344 (3)	C4—H4	0.9300
N1—C27	1.348 (3)	C4—C3	1.394 (3)
F1—C1	1.330 (3)	C19—H19A	0.9700
F2—C2	1.338 (3)	C19—H19B	0.9700
C18—C17	1.401 (3)	C19—C20	1.528 (4)
C18—C27	1.402 (3)	C20—H20A	0.9700
C18—C19	1.526 (3)	C20—H20B	0.9700
C17—C8	1.417 (3)	C15—H15	0.9300
C17—C16	1.495 (3)	C15—C14	1.388 (4)
C8—C7	1.401 (3)	C1—C2	1.376 (4)
C8—C9	1.506 (3)	C25—H25	0.9300
C7—C5	1.496 (3)	C25—C24	1.386 (4)
C5—C6	1.393 (3)	C9—H9A	0.9700
C5—C4	1.395 (3)	C9—H9B	0.9700
C27—C26	1.490 (3)	C9—C10	1.541 (4)
C16—C11	1.399 (4)	C10—H10A	0.9700
C16—C15	1.400 (3)	C10—H10B	0.9700
C26—C21	1.410 (3)	C2—C3	1.363 (4)
C26—C25	1.390 (3)	C12—H12	0.9300
C21—C22	1.396 (3)	C12—C13	1.377 (4)
C21—C20	1.493 (3)	C3—H3	0.9300
C6—H6	0.9300	C24—H24	0.9300
C6—C1	1.374 (3)	C24—C23	1.388 (4)
C11—C10	1.490 (4)	C23—H23	0.9300
C11—C12	1.407 (3)	C13—H13	0.9300
C22—H22	0.9300	C13—C14	1.377 (4)
C22—C23	1.364 (4)	C14—H14	0.9300
			
C7—N1—C27	118.46 (18)	C21—C20—H20A	109.4
C17—C18—C27	117.92 (19)	C21—C20—H20B	109.4
C17—C18—C19	125.17 (19)	C19—C20—H20A	109.4
C27—C18—C19	116.9 (2)	C19—C20—H20B	109.4
C18—C17—C8	118.99 (19)	H20A—C20—H20B	108.0
C18—C17—C16	123.57 (19)	C16—C15—H15	119.7
C8—C17—C16	117.4 (2)	C14—C15—C16	120.6 (3)
C17—C8—C9	118.13 (19)	C14—C15—H15	119.7
C7—C8—C17	118.1 (2)	F1—C1—C6	120.3 (3)
C7—C8—C9	123.6 (2)	F1—C1—C2	118.7 (2)
N1—C7—C8	122.89 (19)	C6—C1—C2	121.0 (2)
N1—C7—C5	114.26 (18)	C26—C25—H25	119.4
C8—C7—C5	122.84 (19)	C24—C25—C26	121.3 (2)
C6—C5—C7	119.4 (2)	C24—C25—H25	119.4
C6—C5—C4	119.0 (2)	C8—C9—H9A	109.7
C4—C5—C7	121.5 (2)	C8—C9—H9B	109.7
N1—C27—C18	123.3 (2)	C8—C9—C10	110.01 (19)
N1—C27—C26	116.34 (19)	H9A—C9—H9B	108.2
C18—C27—C26	120.27 (19)	C10—C9—H9A	109.7
C11—C16—C17	118.0 (2)	C10—C9—H9B	109.7
C11—C16—C15	119.3 (2)	C11—C10—C9	109.1 (2)
C15—C16—C17	122.7 (2)	C11—C10—H10A	109.9
C21—C26—C27	119.1 (2)	C11—C10—H10B	109.9
C25—C26—C27	121.9 (2)	C9—C10—H10A	109.9
C25—C26—C21	119.0 (2)	C9—C10—H10B	109.9
C26—C21—C20	118.1 (2)	H10A—C10—H10B	108.3
C22—C21—C26	118.9 (2)	F2—C2—C1	119.9 (3)
C22—C21—C20	123.0 (2)	F2—C2—C3	119.5 (3)
C5—C6—H6	120.2	C3—C2—C1	120.6 (2)
C1—C6—C5	119.6 (2)	C11—C12—H12	119.9
C1—C6—H6	120.2	C13—C12—C11	120.2 (3)
C16—C11—C10	118.6 (2)	C13—C12—H12	119.9
C16—C11—C12	119.2 (2)	C4—C3—H3	120.3
C12—C11—C10	122.2 (2)	C2—C3—C4	119.4 (3)
C21—C22—H22	119.4	C2—C3—H3	120.3
C23—C22—C21	121.2 (2)	C25—C24—H24	120.4
C23—C22—H22	119.4	C25—C24—C23	119.2 (3)
C5—C4—H4	119.8	C23—C24—H24	120.4
C3—C4—C5	120.4 (3)	C22—C23—C24	120.6 (3)
C3—C4—H4	119.8	C22—C23—H23	119.7
C18—C19—H19A	109.2	C24—C23—H23	119.7
C18—C19—H19B	109.2	C12—C13—H13	119.6
C18—C19—C20	111.98 (19)	C14—C13—C12	120.9 (3)
H19A—C19—H19B	107.9	C14—C13—H13	119.6
C20—C19—H19A	109.2	C15—C14—H14	120.2
C20—C19—H19B	109.2	C13—C14—C15	119.7 (3)
C21—C20—C19	111.3 (2)	C13—C14—H14	120.2

### Hydrogen-bond geometry (Å, º)

**Table:**

*D*—H···*A*	*D*—H	H···*A*	*D*···*A*	*D*—H···*A*
C10—H10*B*···N1^i^	0.97	2.61	3.552 (4)	165
C19—H19*A*···F2^ii^	0.97	2.56	3.111 (4)	116

Symmetry codes: (i) −*x*+1, −*y*+1, −*z*+1; (ii) *x*−1, *y*, *z*+1.
